# The Effect of Soil Mineral Composition on the Compressive Strength of Cement Stabilized Rammed Earth

**DOI:** 10.3390/ma13020324

**Published:** 2020-01-10

**Authors:** Piotr Narloch, Piotr Woyciechowski, Jakub Kotowski, Ireneusz Gawriuczenkow, Emilia Wójcik

**Affiliations:** 1 Faculty of Civil Engineering, Warsaw University of Technology, Al. Armii Ludowej 16, 00-637 Warsaw, Poland; p.woyciechowski@il.pw.edu.pl; 2 Faculty of Geology, University of Warsaw, Żwirki i Wigury 93, 02-089 Warsaw, Poland; j.kotowski@uw.edu.pl (J.K.); i.gawriuczenkow@uw.edu.pl (I.G.); wojcike@uw.edu.pl (E.W.)

**Keywords:** rammed earth, cement stabilized rammed earth, SEM images, mineral composition, compressive strength, sustainable building material

## Abstract

Cemented stabilized rammed earth (CSRE) is a building material used to build load bearing walls from locally available soil. The article analyzes the influence of soil mineral composition on CSRE compressive strength. Compression tests of CSRE samples of various mineral compositions, but the same particle size distribution, water content, and cement content were conducted. Based on the compression strength results and analyzed SEM images, it was observed that even small changes in the mineral composition significantly affected the CSRE compressive strength. From the comparison of CSRE compressive strength result sets, one can draw general qualitative conclusions that montmorillonite lowered the compressive strength the most; beidellite also lowered it, but to a lesser extent. Kaolinite lightly increased the compressive strength.

## 1. Introduction

### 1.1. An Overview of Cement Stabilized Rammed Earth

**Figure 1:**
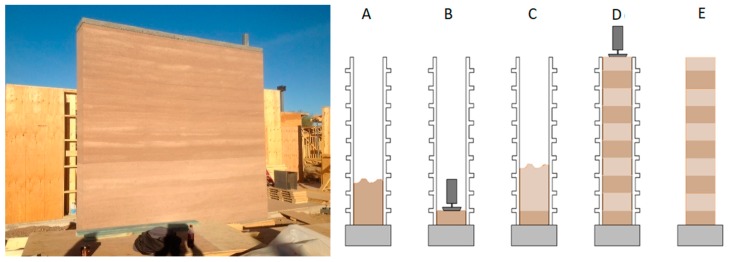
Right: a cement stabilized rammed earth (CSRE) wall. Left: CSRE wall erection scheme. A: the formwork is filled with a layer of moist soil-cement mixture. B: ramming the mixture. C: filling the formwork with another layer of the mixture. D: successive layers of moist mixture are added and compacted. E: the formwork is removed.

Cement stabilized rammed earth (CSRE) is a construction material used to build load bearing walls from locally available soil found under the layer of humus. The technology of erecting walls from CSRE involves dynamic ramming of a moist soil-cement mixture in the formwork. After proper compaction of the layer, the next layers are added until the planned height of the element is reached (Figure 1).

CSRE is a sustainable building material. As a result of the Building Research Establishment Environmental Assessment Method (BREEAM), external building partitions consisting of a CSRE bearing layer obtained the highest A+ rating [1,2]. Portland cement is a frequently used stabilizer added to mixes, which increases the mechanical strength and durability of the material. It is an ideal stabilizer for coarse soils that have less expansive clay minerals. Soils that do not contain clay fractions ease the mixing and cement stabilization processes. The increase of the compressive strength of CSRE from mixes based on these types of soil is more than proportional to the cement added, thanks to which it is often possible to reduce the amount of cement needed significantly to achieve a compressive strength at the intended level [3]. With soils containing clay fractions, lower CSRE compressive strengths are achieved [3]. This is due to the fact that these soils are much more chemically diverse, and the reactions that occur significantly affect the properties of the material [4]. Furthermore, the rate of the increase in compressive strength in soils containing clay fractions is slower compared to mixes consisting of only sand and gravel fractions [4]. The pozzolanic reaction in soils containing clay minerals stimulates a long term increase in compressive strength [4]. The compressive strength of rammed earth from mixtures containing clays can even be reduced by added cement, in particular when less than 5% is added [4].

### 1.2. Factors Affecting the Compressive Strength of CSRE

The compressive strength of CSRE is influenced by a variety of mixture properties and technological features, such as:

- mineral composition of the soil [5];
- density of elements [3,6,7];
- building element shape [5];
- porosity of elements [7];
- particle size distribution of the soil used in the mixture [3,6,8];
- age of the building element [7,9,10];
- mixture compaction method [11];
- moisture content of element during service [6,12,13];
- moisture content of the soil-cement mixture during construction [3,13,14,15,16,17];
- content and type of Portland cement [7,16,17];
- the exposure conditions of the building element [6,18];

Among the technological features mentioned above, focus has been placed on the mineral composition of soil. This is a property of soil that is difficult to predict in a construction setting and is often overlooked in scientific papers. For this reason, the authors decided to verify the significance of the impact of the soil’s mineral composition on the compressive strength of CSRE.

### 1.3. Soil Mineral Composition Effect on the Compressive Strength of CSRE

#### 1.3.1. Introduction

Cohesive soils are composed of clay and non-clay minerals. Among clay minerals, illite is the predominant mineral in the subsurface of Central and Northern European soils (the part that had been glaciated) [19]. Present as well are smectite and kaolinite [19]. There are no differences in the qualitative composition of clay minerals found in the variously aged tills in Central and Northern Europe [19]. Among non-clay minerals, the most commonly found mineral is quartz, which can constitute up to 90% of cohesive soils [19]. Iron compounds such as goethite, siderite, and carbonates (among others, calcite) are also frequently present [19].

#### 1.3.2. Clay Minerals

Clay minerals differ from other minerals in their specific structure. They are hydrated aluminosilicates with a characteristic, layered crystalline structure. One of the layers contains silicate tetrahedrons whose centers are Si^4+^ and are surrounded by four oxygen atoms. These tetrahedrons connect with one another to form the hexagonal arrangement of Si_2_O_2-5_. The second layer consists of octahedrons where Al^3+^ is surrounded by six hydroxyl groups. The layers combine with one other in distinctive arrangements called packets that determine their properties. The most important features of clay minerals include their cation exchange capacity and their ability to absorb water [17]. About 15 minerals are ordinarily classified as clay minerals, and these belong to three main groups: kaolin, illite, and smectite [20]. These groups of minerals are the most commonly occurring clay minerals in soils [21,22,23].

Kaolinite is the most widespread mineral in the kaolin group. Its structure consists of one tetrahedral layer and one octahedral layer. The hydrogen bond is strong, and crucially, the intra-lamellar spacing is very small; hence, it is extremely difficult to separate the layers, and as a result, kaolinite is relatively stable and water less able to penetrate between the layers; thus, it exhibits relatively little swell on wetting [24,25].

Illite is the most common mineral from the illite group. Its structure consists of one octahedral layer and two tetrahedral layers. It is characterized by a rigid structure, which is due to the presence of K^+^ cations in the spaces between the packets. These cations connect negatively charged surfaces, causing the illites not to swell as much as smectites [26]. Illites are classified as non-swelling or low swelling minerals [26].

In the smectite group, the most widespread minerals are beidellite and montmorillonite. These minerals, like illite, are made of two tetrahedral layers and an octahedral layer bound between them. The thickness of this packet is about 1 nm (10 Å). Montmorillonites are characterized by their high water absorption, which results mainly from their structure and from the existence of interlayer cations. Smectites differ from illites in that they have a labile structure, i.e., interlayer spaces may expand [27].

**Figure 2:**
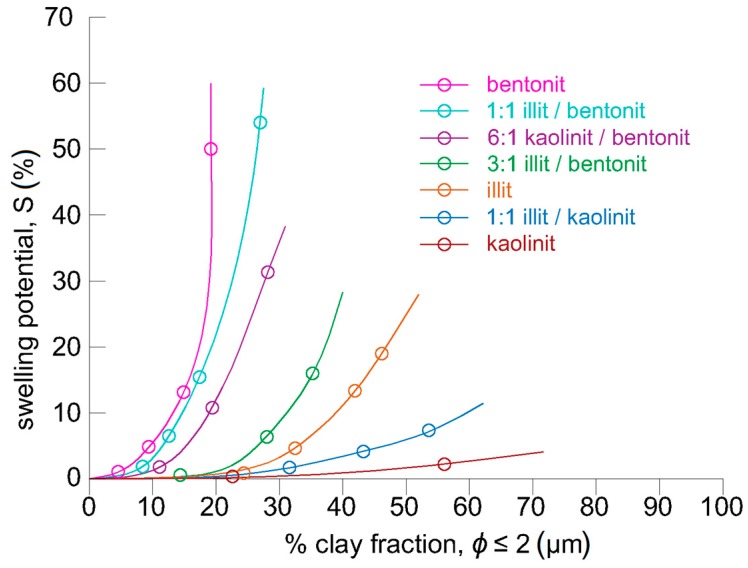
Relationship between swelling potential and clay fraction percentage (after Seed et al.) [28].

The swelling potential (Figure 2) of the minerals described above indicates that soils containing even small amounts of smectites will swell intensively, while soils with illite or kaolinite will not. In the opinion of the authors, it is therefore not possible to treat the clay faction in soil as always having a detrimental effect on the compressive strength of CSRE. This approach would indicate that soil without clay fractions (e.g., sand with cement) will not necessarily have a higher compressive strength compared to soil containing clay fractions.

**Figure 3:**
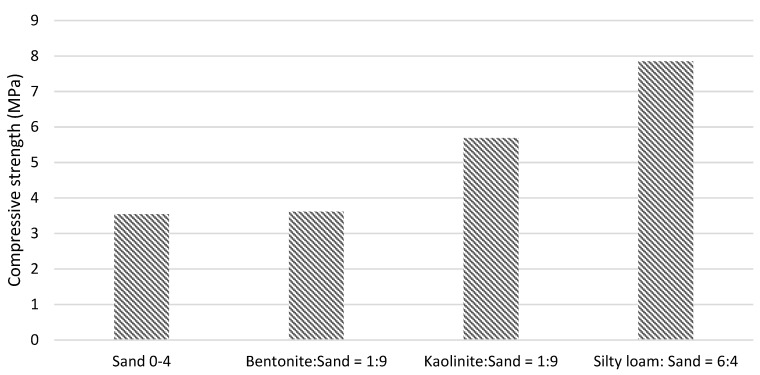
Compressive strengths of adobe bricks with the addition of 6% cement. Based on [4].

The studies described in [4] showed that soil mixes containing a large quantity of kaolinite (>10%) obtain higher compressive strengths compared to sand and cement mixes (Figure 3). In turn, soil mixes with montmorillonite obtain lower compressive strengths compared to mixes with kaolinite [29]. Interestingly, mixes with expansive bentonite have obtained similar compressive strengths to mixes with sand alone. Test results also indicate that granulation has a key impact on compressive strength. The CSRE soil mix composed of 60% silty loam and 40% sand obtained a higher strength than the rest.

#### 1.3.3. Non-Clay Minerals

Quartz, SiO_2_, is among the most common minerals found in the Earth’s crust. It occurs in many diverse varieties, including in the form of coated grains in sands, as well as in cohesive soils where it forms aggregates and microaggregates with clay minerals. It is characterized by a high hardness (seven on the Mohs Hardness Scale) and a density of 2.65 g/cm^3^. These properties increase the compressive strength of CSRE [5]. It is a mineral that does not react with water.

Another common mineral is goethite, FeO(OH). It is a mineral found in sedimentary rocks, usually occurring in compact, granular, and earthy clusters. It is less hard than quartz (5.5 on the Mohs Hardness Scale), and because of its high iron content (62.8%), it has a notably high density (4–4.4 g/cm^3^). This may influence the bulk density of a CSRE element. An important feature of goethite is its color (usually, various shades of brown), often having an effect on the color of CSRE. Siderite, FeCO_3_, occurs mainly in a compact, earthy, or very fine crystalline cluster form. It has a lower hardness than goethite (4–4.5 on the Mohs Hardness Scale), and because it has a smaller iron content (48.3%), it therefore has a smaller density (3.7–3.9 g/cm^3^). It usually has a dark-yellow, orange color. Like the non-clay minerals mentioned above, it does not absorb water. Goethite and siderite in the presence of water are stable at temperatures from 0 to 100 °C. This means that they will not react with water [30].

Calcite, CaCO_3_, is an important rock forming mineral. It creates very diverse forms of clusters: granular, compacted, and fibrous. This mineral is the main component in sedimentary limestone rocks, both organic and chemical in origin. In soils, calcite plays a critical role, affecting a number of their properties, also acting structurally. It has a relatively low hardness (3.0 on the Mohs Hardness Scale), similar to clay minerals. The density of calcite varies from 2.6 to 2.8 g/cm^3^. It does not absorb water, but it easily dissolves in acid (e.g., carbonic acid).

### 1.4. Cement Stabilization

As mentioned above, each mineral behaves differently in contact with water, which in turn has an effect on cement binding. Cement stabilization for CSRE is best suited for soils with less expansive minerals. Unlike concrete, where early stabilization is caused by cement, in CSRE, there is a long lasting curing of the material thanks to which the strength continues to grow past 28 days [4,31,32].

## 2. Materials and Methods

### 2.1. Materials

**Figure 4:**
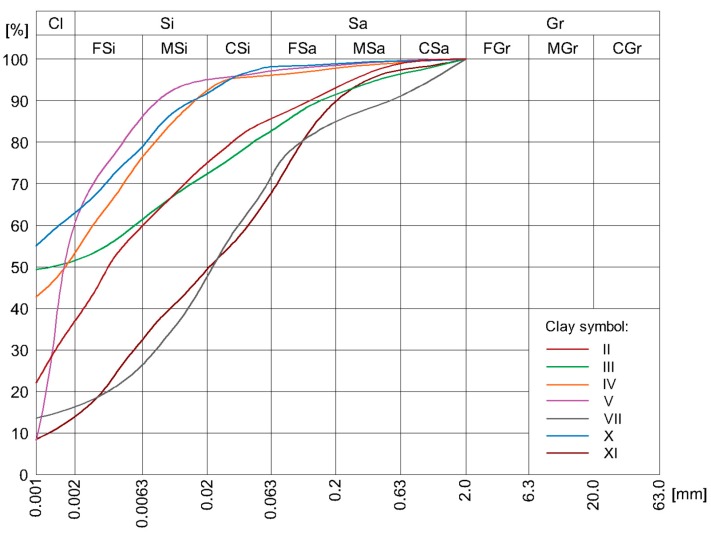
Granulation curves of selected clays.

The study examined the relationship between the mineral composition of soil-cement mixtures and the compressive strength of the CSRE. Soil mixtures with the same particle size curves were prepared. Accordingly, soil mixtures consisting of sieved sand and gravel fractions, as well as seven different loams were used. In terms of mineral composition, sand was composed of pure quartz. Gravel fractions were composed of quartz (75% by mass) and carbonate crumbs (25% by mass). The clays used in the study differed in both mineral composition (Table 1), as well as granulation (Figure 4).

**Figure 5:**
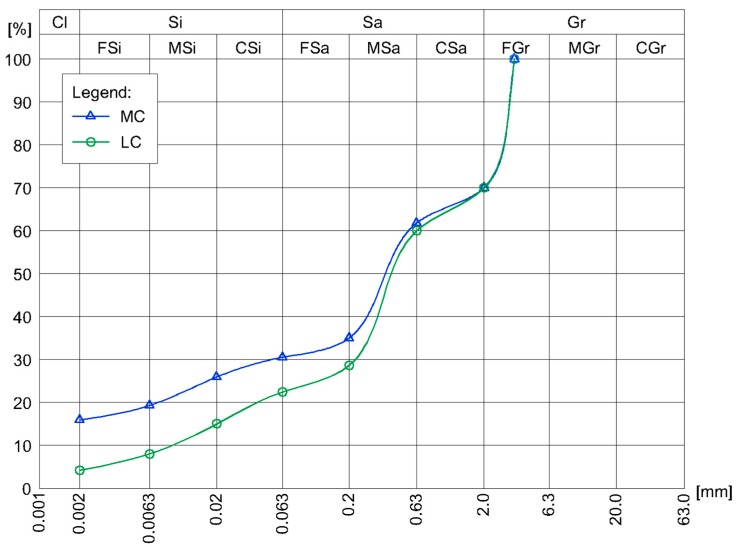
Particle size distribution of soil mixtures used in the CSRE compressive strength tests.

By adding an appropriate amount of dry ingredients (clays, as well as sand and gravel fractions; Table 2), mixtures were obtained with granulation curves as shown in Figure 5. These mixtures simulated two potential soils that could be used as the main component of CSRE. These mixtures differed first and foremost in the proportion of clay fractions. The MC mixture consisted of a 16% clay fraction, 15% silt fraction, 39% sand fraction, and 30% gravel fraction. Meanwhile, the LC mixture consisted of a 4% clay fraction, 18% silt fraction, 48% sand fraction, and 30% gravel fraction. The LC mixtures were designed with clays II, VII, and XI, while the MC mixtures with clays III, IV, V, and X.

Portland cement is a frequently used stabilizer for rammed earth mixtures. Due to its hydration with water, it significantly increases the compressive strength of CSRE. In [3], it was shown that for rammed earth samples (from soil mixtures containing 30% clay and silt, 40% sand, and 30% gravel, with optimum humidity, seasoned for 28 days), by adding 9% cement, compressive strength increased from about 2.68 MPa to about 9.30 MPa. Cement CEM I 42.5 R (Table 3) was added to these mixtures, in the amount of 6% and 9% by mass of the remaining dry ingredients. These values were chosen based on the authors’ own previous study, which found that 6% cement ensured an adequate compressive strength for the CSRE [3,5,30], while 9% cement was needed in regards to durability [33,34,35,36,37].

To the 14 soil-cement mixtures (Table 4) that were obtained in this way, water was added, ensuring that the optimum moisture content (OMC) of the mixture was achieved, i.e., the moisture at which compaction by ramming achieved the maximum value of dry density (the method of determining the OMC is given in Section 2.2.2). The OMC is the water content at which a soil can be compacted to the maximum dry density by a given compactive effort [38]. Therefore, OMC is a direct measure of CSRE workability.

The workability of the CSRE mixture is the ability to accurately fill molds while maintaining its uniformity and tightness and the use of a specific compaction method. This is an important feature of the mixture, affecting the mechanical strength and durability of the material, as well as the work needed to erect vertical partitions [4]. Similar to the concrete mixture, the workability of the CSRE mixture will be influenced, among others, by the amount of water added to the mixture, the grain size of the soil used for the mixture, the shape and texture of the aggregate grains, the content of cement, or other stabilizers, admixtures, and additives used [39].

Proper workability of the CSRE mixture is a condition for its effective compacting. In CSRE technology, compaction occurs by ramming a moist soil mixture in the formwork. The way the mixture is compacted plays an important role in shaping the appropriate compressive strength of the material [4]. For rammed earth, no consistency test methods shall be used [4,14,16]. In this case, the measure of workability is the OMC [40]. For this reason, the assessment of whether the mixture has adequate workability will be affected by both the ramming energy and the thickness of the compacted material layers.

The OMC for a specific mixture design is based on the maximum dry density at which maximum compressive strength is achieved [3]. Determining the OMC of the soil-cement mixture guaranties the proper workability. It is one of the key issues in CSRE technology. The OMC for lower clay content mixes (LC) was 7% and for higher clay content mixes (MC) was 8%, regardless of the mineral composition and cement addition in the mixtures.

#### Preparation of Samples

**Figure 6:**
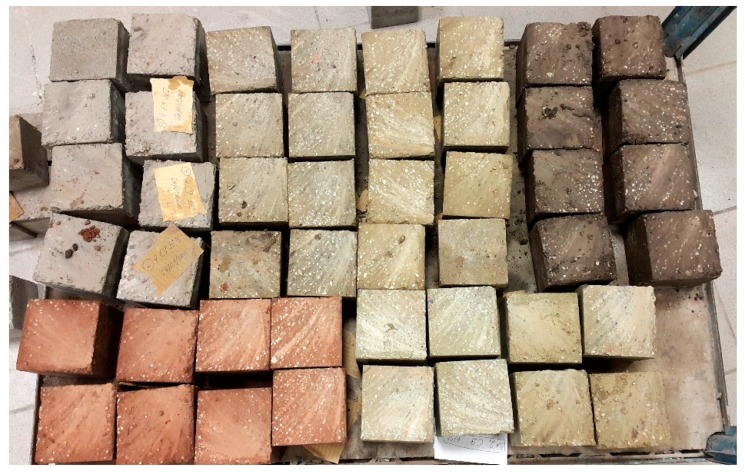
Part of the CSRE samples on which the compressive strength tests were made.

Clay, sieved sand fractions, and gravel were mixed together in a dry state. Then, cement was added and everything mixed again. Finally, water was poured. After reaching a uniform consistency, 100 × 100 × 100 mm cubic samples were formed. The formation of the samples was carried out by ramming the mixture in three equal layers using a 6.5 kg rammer. The samples were formed by freely lowering the rammer from a height of 30 cm to the surface of the moist mixture filled in the form. Samples were preliminarily formed at a height of 105 mm. The day before planned compressive strength testing, they were cut to a height of 100 mm (Figure 6).

The removed 5 mm slabs slowly dried to an air-dry state (20 °C and 50% RH). In this state, polished thin sections for SEM analyzes were made. Samples were not artificially dried in dryers at any preparatory stage. Therefore, the process of the preparation of samples for SEM testing did not significantly affect the observed cracks, which, according to the authors, were largely caused by natural, slow drying, such as occurs in construction conditions.

Since the rammed earth keeps the layered structure, the samples in the compressive strength test were loaded in the direction of ramming. For each series, 10 samples were prepared. The samples were demolded after 24 h. Then, they were cured for 28 days in a condition of a high relative humidity of 95% (±2%) and a temperature of 20 °C (±1 °C).

### 2.2. Methods

#### 2.2.1. The Method for Determining the OMC of Soil-Cement Mixtures

The OMC of a soil-cement mixture is dependent on the particle size distribution of the soil, cement content in the mixture, and mineral composition of the soil. With small amounts of clay fractions, the mineral compositions of the mixtures would not change much. Soil particle size and cement addition have more impact [32]. The method and energy of ramming would also have an effect on this value. For this reason, a method to determine the optimum moisture content of soil mixtures used for preparing rammed earth samples for compressive strength test proposed in [39] was used. This method is based on rammed earth soil compaction method given by NZS 4298: 1998 [40]. According to it, the moisture of a soil mixture for which the maximum dry density is obtained should be determined using the ramming method such as the samples that were compacted. This in turn should reflect the nature and force of the dynamic compaction used for building rammed earth walls. The paper adopts the method of ramming samples described in NZS 4298: 1998 [40]. The maximum dry density of samples from the mixtures was determined for this compaction method.

The components of the soil mixture were dried to a constant mass and then mixed with each other. Then, water was added to obtain the OMC. The mold was weighed before measuring the density of the mixture. Next, ramming was carried out as specified in p. 2.1.1. After completion, the excess soil mixture above the mold surface was removed and then weighed. Then, the mass of the sample was calculated. Knowing the volume of the mold, the bulk density of the soil mixture ρ was calculated for a given moisture:

(1) $$\rho =\frac{m_{m}}{V}$$

where:

$m_{m}$: sample soil mass in (kg)

V: volume of the sample in $(m^{3}$)

Knowing the bulk density of the soil mixture, ρ, the dry density, $\rho_{d}$, was calculated:

(2) $$\rho_{d}=\frac{100\rho }{100+w}$$

where:

ρ: bulk density of soil mixture $\left(\frac{\mathrm{kg}}{m^{3}}\right)$

*W*: moisture of sample mixture (%)

For every soil mix of a given moisture content, measurements of dry density were carried out on three samples; the arithmetic mean of the measurements was taken as the value of the dry density.

#### 2.2.2. Methods for Determining the Granulation of Loams

In order to determine the grain size distribution of each loam, an aerometric analysis was carried out for each of them in accordance with standard PN-B-04481:1988 [41]. In this analysis, the relationship between the falling speed of particles in water and their diameter was used to determine the granulometric composition. Aerometric analysis was used in determining the content of soil particles with equivalent diameters less than 0.06 mm in cohesive soils [20].

#### 2.2.3. SEM Methodology

The SEM-EDS analysis was carried out in order to identify the mineral composition precisely and to present the texture of the samples in the micro-area. Electron microscopy combined with X-ray microanalysis gave the possibility of precise identification of the chemical composition and thus also the mineralogical of the samples tested. The SEM method allowed the observation of the surface of the sample at high magnifications, up to 100,000×. High magnifications allowed observing clusters and aggregates of clay minerals and the clay-silt matrix between the mineral framework.

Observations and analyses of aggregate minerals were made by scanning electron microscopy (SEM) at the National Multidisciplinary Laboratory of Functional Nanomaterials at the Faculty of Geology, University of Warsaw. Thin carbon sputtered (20 nm) sections were examined using the ZEISS Sigma VP FE-SEM (scanning electron microscope with field emission, Carl Zeiss AG, Oberkochen, Germany) equipped with a dispersive X-ray spectrometer (EDS). To record the spectra, the 20 kV electron beam’s acceleration voltage was used. To obtain the maximum current, required for high contrast imaging and element mapping, a 120 µm aperture was chosen, as well as an optional, high-current Zeiss mode. Mappings (spatial arrangement) of selected chemical elements were carried out at the above mentioned conditions and a time of counting of 10 min/frame.

#### 2.2.4. CSRE Compressive Strength Test

Compressive strength tests on CSRE samples were carried out in a testing machine with two test modules: with 0–3000 kN and 0–250 kN ranges of measurement, with a measurement error of less than 1%. The tests were carried out in a similar fashion to the test method for the compressive strength of concrete samples given in standard EN 12390-3 [42]. The only difference was the method of placing samples in the strength testing machine. Rammed earth, laid and compacted in layers, retained this layered structure in both a monolithic wall and in molded samples. Due to the material characteristics, as well as the forming method, it was considered representative of rammed earth that the method of testing the compressive strength was by loading the sample in the direction of its formation.

#### 2.2.5. Moisture Content of CSRE Samples after the Curing Period

After testing the compressive strength, the crushed fragments of one CSRE sample were collected from each of the 14 tested series. The fragments were weighed and then dried to a dry mass at 105 °C. Mass measurements were done every 24 h. When two consecutive measurements differed by less than 1 g, the elements were considered to have reached their dry mass. Knowing the dry mass of crushed CSRE sample, the moisture content of the CSRE (w) was calculated.

(3) $$w=\frac{m_{humid}-m_{dry}}{m_{dry}}$$

where:

$m_{humid}$: mass of the humid crushed CSRE sample (kg);

$m_{dry}$: mass of the dry crushed CSRE sample (kg).

## 3. Results

### 3.1. CSRE Compressive Strength and Moisture Content

**Figure 7:**
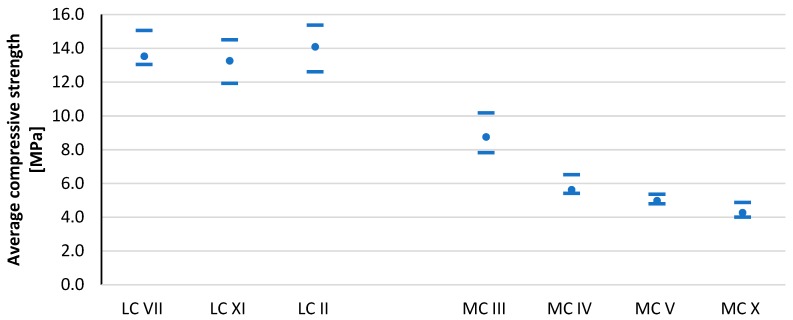
Compressive strength of the CSRE sample series with 9% cement content. Horizontal lines mark the minimum and maximum value in the sample series, while the round points mean the average value.

**Figure 8:**
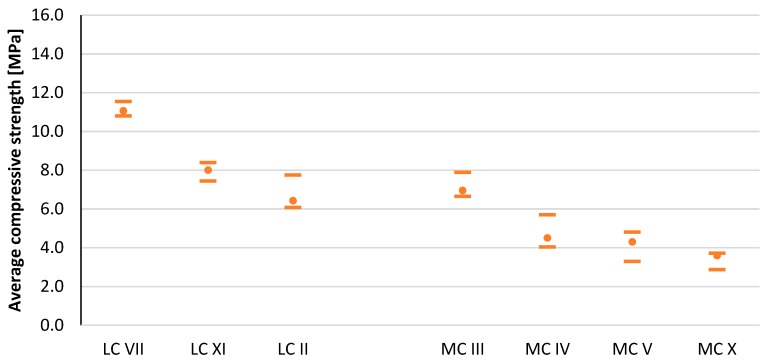
Compressive strength of the CSRE sample series with 6% cement content. Horizontal lines mark the minimum and maximum value in the sample series, while the round points mean the average value.

**Figure 9:**
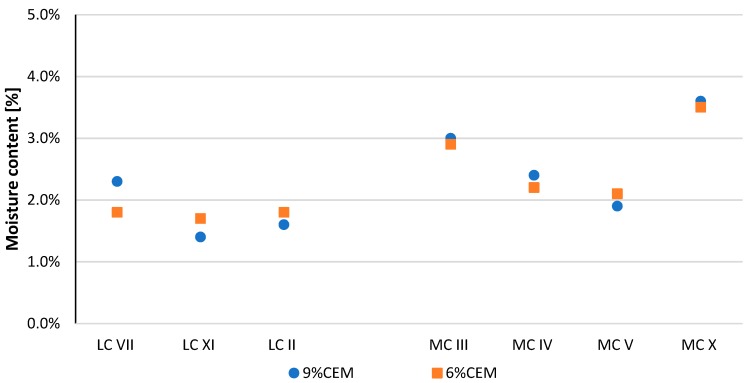
Average moisture content of each CSRE sample series during the compressive strength tests.

The compressive strength results after the curing period for all tested CSRE series are shown in Figure 7, Figure 8, and Table 5. The average moisture content for all series is shown in Figure 9 and Table 5. By comparing the results from the LC and MC series, it can be seen that granulation had a key influence on compressive strength. The average moisture contents of the MC sample series (with more clay) were higher than those of the LC sample series (with less clay). As expected, significant differences were observed between the moisture content of samples with the same granulation, but different mineral composition.

The MC III mixture contained the least amount of clay minerals. Besides beidellite and kaolinite, the most likely mineral to comprise the clay fraction was calcite. For this reason, CSRE made from this mixture had the highest compressive strength of those in the CSRE group containing a high clay content. Mixtures MC IV and MC V were characterized by their almost identical mineral composition; hence, they obtained similar compressive strength results. These almost identical results confirmed that the mineral composition affected the strength of the samples. The lack of swelling minerals (smectites, i.e., beidellite and montmorillonite) in the MC IV and MC V mixes resulted in a noticeably higher compressive strength compared to the MC X mix. The MC X mixture was the only mix containing montmorillonite, a highly swelling mineral with high hydrophilicity. For this reason, CSRE samples from this mixture had the highest moisture content at the time of testing and consequently the lowest compressive strength. The MC III mix contained 6.6% beidellite, which was also a swelling mineral. For this reason, this mixture was characterized by a higher moisture content, compared to mixes MC IV and MC V.

**Figure 10:**
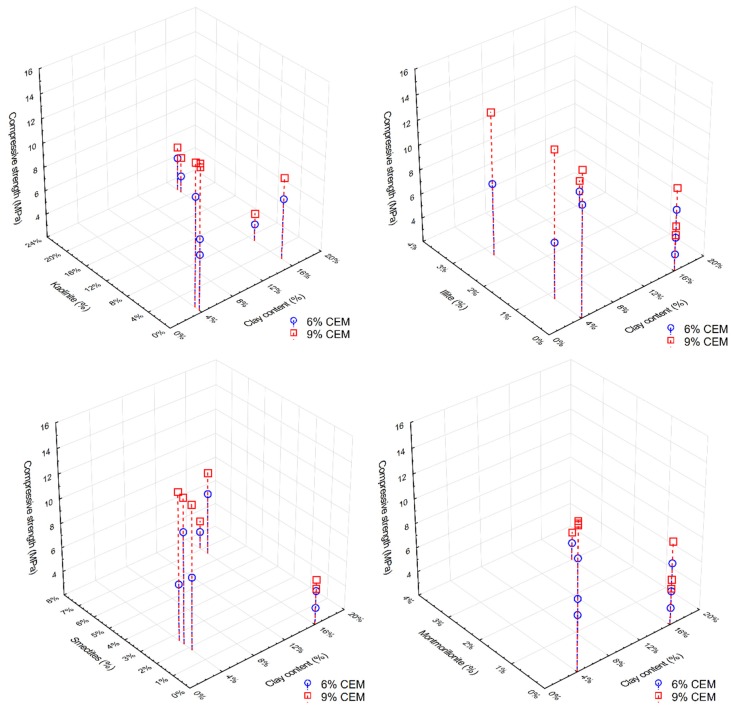
Compressive strength in comparison to the clay fraction content and the amount of each clay mineral: kaolinite (top left), illite (top right), smectites (bottom left), and montmorillonite (bottom right).

Figure 10 presents the compressive strength results of all tested CSRE series, depending on the clay fraction content and the content of individual clay minerals in the soil mixture. From the graph showing the relationship between the CSRE compressive strength and kaolinite content, one could conclude that as the kaolinite content increased, the compressive strength decreased. However, comparing the results of mixtures MC IV, MV V, and MC X showed the opposite result, that with more kaolinite, there as a slightly higher compressive strength. The high compressive strength of the CSRE from the MC III mix (low kaolinite mix) was due to the high calcite content. The impact of illite is difficult to determine; the graph revealed that the other minerals affected the result. The CSRE series containing montmorillonite obtained the lowest compressive strength. The higher compressive strength of the samples from the MC III mixture compared to samples from mixtures containing a higher (16%) clay fraction content (MC IV, MC V, and MC X) did not result from the beneficial effect of calcite, but from a lower content of clay minerals in the clay fraction in the MC III mixture.

Calcite is not a clay mineral and does not swell at all. Even kaolinite swells more than calcite. Hence, the highest compressive strength of the samples from the MC III mixtures resulted. Calcite, however, undergoes weathering processes, which also has a destructive effect on the CSRE compressive strength. This also explains why the LC II mixture obtained the worst compressive strength in the group of mixtures with a low clay fraction content (4%, LC).

**Figure 11:**
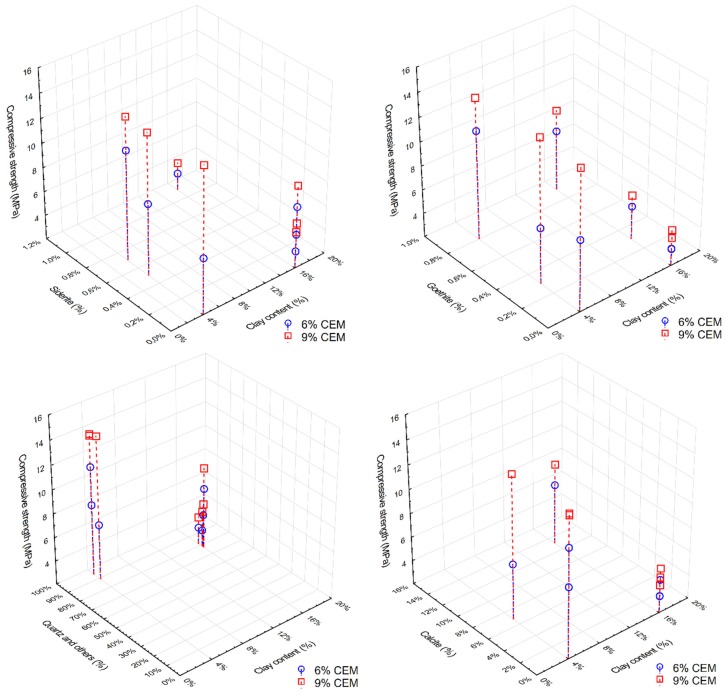
CSRE compression strength in comparison to the clay fraction content and on the amount of certain non-silicate minerals: goethite (top left), siderite (top right), quartz (bottom left), calcite (bottom right).

Figure 11 shows the compressive strength results of all tested CSRE series, depending on the clay fraction content and the content of certain non-clay minerals in the soil mixture. For CSRE with a higher clay content (16%), a significant increase in compressive strength was observed along with an increase in the calcite content. It is worth mentioning in the group with the lower clay fraction content that the opposite was true. CSRE samples made from high clay content mixtures, which had a high percentage of calcite and were stabilized with 6% cement, produced comparable compressive strength results to samples from mixtures with low clay content that did not contain calcite. The results for series with mixtures containing a 9% addition of cement differed significantly. Samples from these two series obtained the largest discrepancies in the results.

Goethite’s low influence was noticeable in mixtures with high clay content and was also beneficial. The effect of siderite on compressive strength was unfavorable and visible with high clay contents. The influence of quartz was favorable, which resulted from the fact that the clay content was lower, and the composite became similar to cement concrete. The effect of calcite was debatable and depended on the form in which it occurred.

### 3.2. SEM Image Analysis

**Figure 12:**
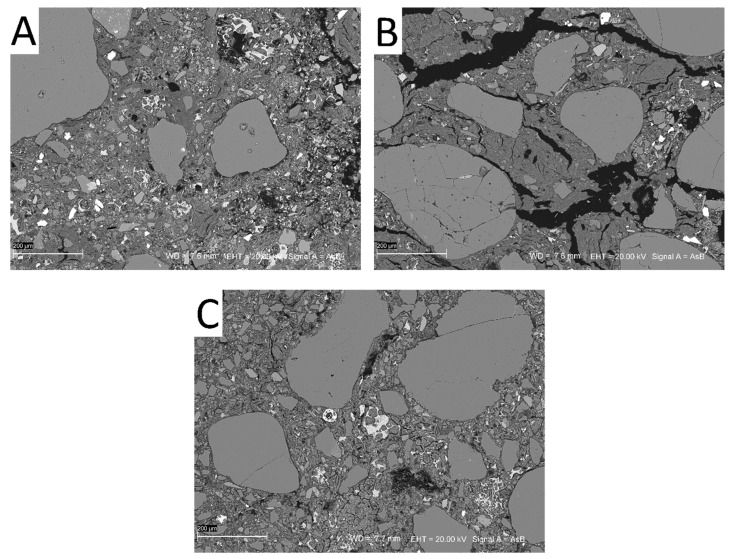
SEM images of samples MC V 9% CEM (**A**), MC X 9% CEM (**B**), and MC III 9% CEM (**C**). Symbols in the figures: WD—working distance; EHT—accelerating voltage; AsB—backscattered electrons (BSE) detector.

Comparing SEM images of CSRE samples from mixtures MC V 9% CEM (Figure 12A) and MC X 9% CEM (Figure 12B) showed that the MC X 9% CEM sample had more cracks. This may be due to the presence of montmorillonite, which when absorbing water, caused swelling.

Clusters of unreacted cement stood out as light “grains” against other mineral grains or mixture of clay minerals constituting the dark grey background of the sample. Unreacted cement, in addition to the characteristic brightness threshold, also had a relatively characteristic texture, it occurred as a filling in the background of the sample, bonding clusters of clay minerals. In contrast, iron oxides, barite, or other rock forming minerals occurred as single grains embedded in clay (the details of SEM image analysis based on the brightness threshold supported by point EDS analyzes of the specific grains with distinctive shades of gray are included in Appendix A of the article).

**Figure 13:**
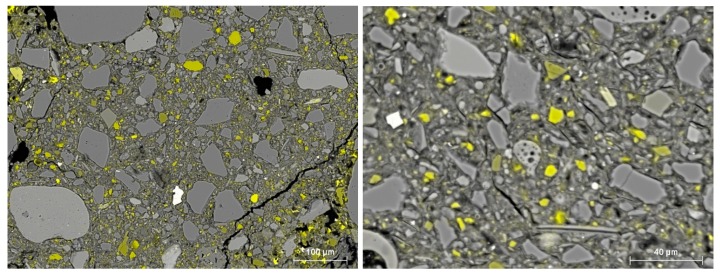
BSE image of a sample of MC III 9% CEM, showing the occurrence of calcium. The numerous carbonate grains are marked in yellow.

**Figure 14:**
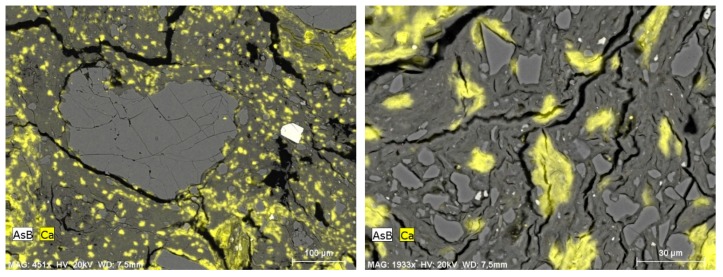
BSE image of a sample of MC X 9% CEM. Visible yellow calcium patches are not carbonates, but clay minerals rich in Ca.

In both the MC III 9% CEM (Figure 12C) and MC V 9% CEM (Figure 12A) samples, unreacted cement (white areas) could be seen, but more so in the MC V 9% CEM sample. Higher amounts of unreacted cement reduced the CSRE’s compressive strength. Figure 13 and Figure 14 show BSE images of samples with a high clay content (MC) mixture with the addition of 9% cement. The yellow indicates visible calcium compounds in the form of limestone grains in MC III (Figure 13) and calcium compound rich clay minerals in MC X (Figure 14). A high occurrence of calcium compounds in clay minerals (including montmorillonite) was correlated with the occurrence of a large number of microcracks in the CSRE structure (Figure 14). This can be treated as a reason for the low compressive strength results obtained by the MC X series.

**Figure 15:**
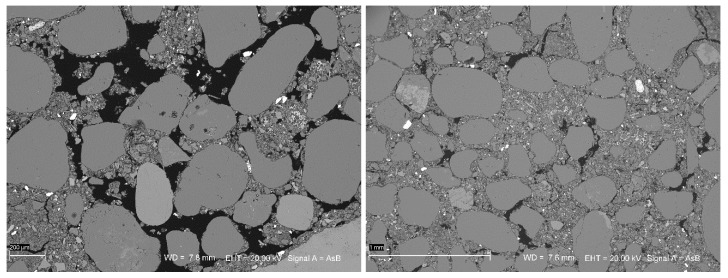
SEM images of samples LC XI 6% CEM (left) and LC XI 9% CEM (right).

When analyzing the SEM images of samples from a low clay fraction mixture (Figure 15), numerous cracks, gaps, and pores were clearly visible as in the image of the LC XI 6% CEM sample. The image of the sample from the LC XI 9% CEM mixture showed that the granular skeleton was compact, with a significantly reduced number of gaps compared to the sample with 6% cement. This resulted in a significant difference in the obtained compressive strength (over 5 MPa). The compressive strength of the MC XI series with a 6% cement addition, amounting to approximately 8 MPa, allowed the material to be used as a structural component. On the other hand, the SEM image analysis suggested that samples with 6% cement may have insufficient durability, especially in climates where CSRE partitions may be exposed to cyclical freezing and thawing, as well as cyclical wetting and drying. Even at the optimum moisture content of the LC 6% CEM (7%) mixture, it remained poorly workable and, as a result of its compaction, produced a highly porous structure with a low compressive strength.

## 4. Conclusions

The research presented in this article showed that mineral composition has a significant impact on the compressive strength of CSRE. For this reason, the authors suggested that all research articles on rammed earth should include mineral composition, along with other key soil properties such as granulation, moisture content, and stabilizers. The authors’ intention was not to determine the effect of individual minerals on the compressive strength of CSRE samples, due to the fact that in nature, such monomineral soils are very rare. Another problem is the interaction between individual minerals. From the comparison of the CSRE compressive strength result sets, one can draw general qualitative conclusions:

- Montmorillonite, as a hydrophilic mineral, set the moisture balance between the sample and the environment at a higher level than for samples not containing this mineral. The result was a higher moisture content at the time of testing of CSRE samples containing montmorillonite, and consequently a lower strength.
- Beidellite slightly reduced the compressive strength.
- Illite did not explicitly affect the compressive strength.
- Kaolinite, as a non-swelling mineral, slightly increased the compressive strength.
- Due to the low percentage of non-clay minerals with the exception of quartz, it was difficult to clearly assess their impact on compressive strength.
- The higher compressive strength of the samples from the MC III mixture compared to samples from mixtures containing a higher (16%) clay fraction content (MC IV, MC V, and MC X) did not result from the beneficial effect of calcite, but from a lower content of clay minerals in the clay fraction in the MC III mixture. Calcite is not a clay mineral and does not swell at all. Even kaolinite swells more than calcite. Hence, the highest compressive strength of the samples was from the MC III mixtures. Calcite, however, undergoes weathering processes, which also has a destructive effect on the CSRE compressive strength. This also explained why the LC II mixture obtained the worst compressive strength in the group of mixtures with a low clay fraction content (4%, LC).

The study also confirmed that soil granulation has a primary effect on CSRE compressive strength. For mixtures stabilized with cement, it is recommended to use soils with a low clay fraction.

## Figures and Tables

**Table 1:** Mineral composition of each loam (%).

Loam Symbol	Montmorillonite	Beidellite	Kaolinite	Illite	Goethite	Siderite	Calcite	Organic Substance	Quartz and Others
II	-	8.6	1.3	2.8	1.0	-	22.7	0.6	63.0
VII	-	8.6	4.7	-	3.0	2.8	-	1.3	79.6
XI	-	8.9	3.1	29.8	-	4.1	-	-	54.1
III	-	21.7	6.1	-	3.0	-	43	0.3	25.9
IV	-	-	72.7	-	1.0	-	-	0.4	25.9
V	-	-	79.8	-	-	-	-	0.5	19.7
X	10	18.1	27.2	11.3	-	4.3	-	1.4	27.6

**Table 2:** Percentage mass compositions of each soil mixture.

Particle Size Distribution	Loam Symbol	(%) Mass of Loam	(%) Mass of Silt and Sand Fractions						(%) Mass of Gravel
			FSi	MSi	CSi	FSa	MSa	CSa	FGr
			(0.002; 0063) (mm)	(0.0063; 0.02) (mm)	(0.02; 0.063) (mm)	(0.063; 0.2) (mm)	(0.2; 0.63) (mm)	(0.63; 2.0) (mm)	(2.0; 4.0) (mm)
LC	II	30.1	0.0	1.9	1.0	0.0	27.7	9.2	30.0
	VII	25.8	3.0	1.5	0.3	3.2	28.4	7.7	30.0
	XI	11.3	3.0	5.3	5.3	5.8	29.4	9.9	30.0
MC	III	30.9	3.8	1.6	0.2	1.8	24.6	7.1	30.0
	IV	29.8	0.0	0.2	2.3	4.0	25.7	7.9	30.0
	V	26.3	0.1	2.7	2.9	4.1	25.8	8.1	30.0
	X	25.2	2.9	1.8	1.8	4.3	25.9	8.1	30.0

**Table 3:** Chemical composition of Portland Cement CEM I 42.5 R.

Constituent	% by Weight
SiO_2_	20.6
Al_2_O_3_	6.2
Fe_2_O_3_	2.9
CaO	64
MgO	1.4
Na_2_O	0.7
SO_3_	2.5
N_2_O	0.05
Cl^-^	0.04
Loss of ignition	2.5

**Table 4:** List of mixtures from which CSRE samples were prepared.

Mixture Symbol	Particle Size Distribution Curve	Loam Used in the Mixture	Water Content (%)	Cement Addition (%)
LC II 6%		II	7	6
LC II 9%				9
LC VII 6%	LC	VII		6
LC VII 9%				9
LC XI 6%		XI		6
LC XI 9%				9
MC III 6%	MC	III	8	6
MC III 9%				9
MC IV 6%		IV		6
MC IV 9%				9
MC V 6%		V		6
MC V 9%				9
MC X 6%		X		6
MC X 9%				9

**Table 5:** Mineral compositions of soil mixtures given in percentages. The table also gives the percentage of water and CEM I 42.5 R cement additions and the average obtained compressive strength of CSRE samples in each series.

Mixture Symbol	Montmorillonite (%)	Beidellite (%)	Kaolinite (%)	Illite (%)	Goethite (%)	Siderite (%)	Calcite (%)	Organic Substance (%)	Quartz and Others (%)	Cement Addition (%)	Water Content (%)	Compressive Strength (MPa)
LC II 6%	0.0	2.6	0.4	0.8	0.3	0.0	6.8	0.2	88.9	6	7	6.43
LC II 9%										9		14.09
LC VII 6%	0.0	2.3	1.2	0.0	0.8	0.7	0.0	0.3	94.6	6	7	11.07
LC VII 9%										9		13.54
LC XI 6%	0.0	1.8	0.4	2.7	0.0	0.5	0.0	0.0	94.6	6	7	8.01
LC XI 9%										9		13.26
MC III 6%	0.0	6.6	1.9	0.0	0.9	0.0	13.1	0.1	77.3	6	8	6.96
MC III 9%										9		8.76
MC IV 6%	0.0	0.0	21.8	0.0	0.3	0.0	0.0	0.1	77.8	6	8	4.51
MC IV 9%										9		5.63
MC V 6%	0.0	0.0	21.1	0.0	0.0	0.0	0.0	0.1	78.7	6	8	4.30
MC V 9%										9		5.45
MC X 6%	3.0	4.1	6.9	2.9	0.0	1.1	0.0	0.4	81.7	6	8	3.60
MC X 9%										9		4.27
Legend (%)	0.1–1	1–5	5–10	10–25	>25							

**Figure A1:**
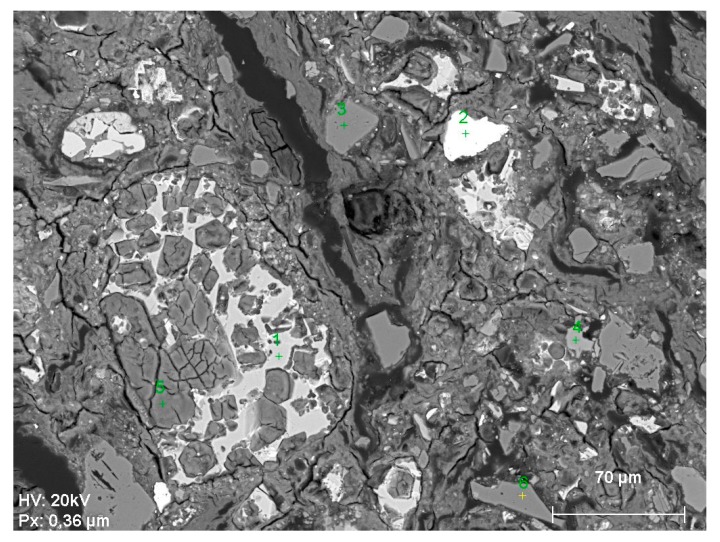
BSE photo of the sample along with EDS analysis locations.

**Figure A2:**
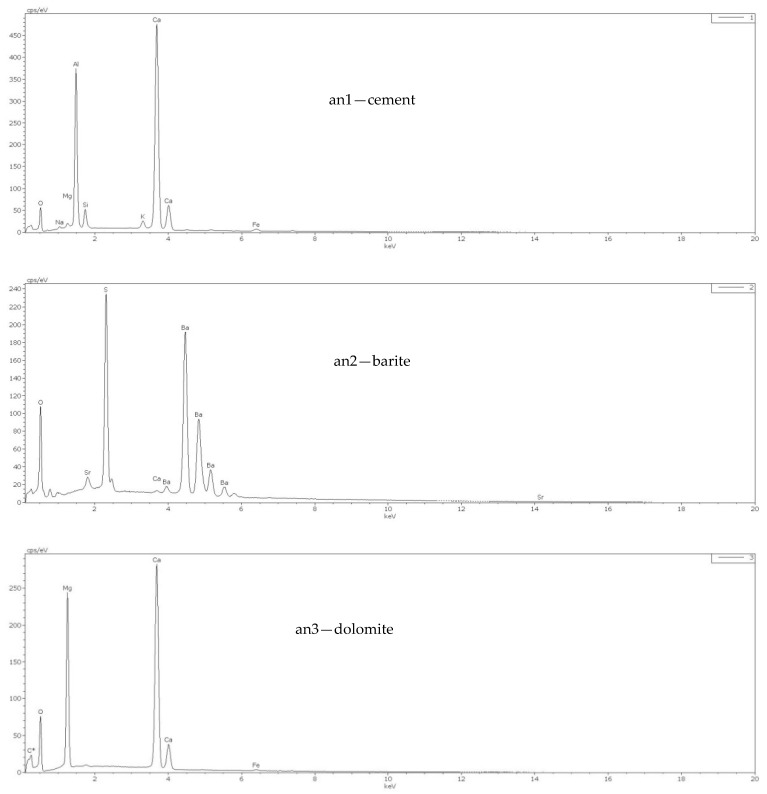

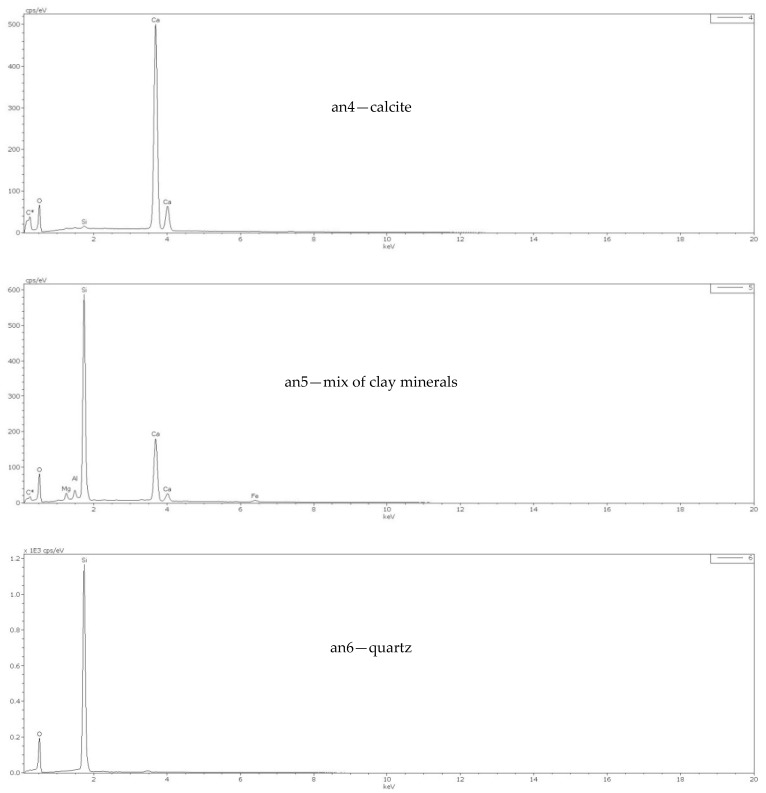
EDS spectra of grains with different shades of grey indicate different chemical composition.

## Appendix A

Figure A1 is a BSE photo of the sample along with energy-dispersive X-ray spectroscopy (EDS) analysis locations (an1–an6). Figure A2 shows full EDS spectra. Measurements were done with 20 kV voltage and point analysis with 30 s counting.

When analyzing a sample in SEM using a BSE (backscattered electrons) detector, the image in various shades of gray, with so called, material contrast was received. This means that a material made of a substance with a higher electron density (in most cases, with a higher atomic number) will have a lighter shade than a material made of a substance with lighter elements. It is often the case that mineral grains with different chemical compositions have a very similar shade of gray (Figure A2, an3—dolomite, an4—calcite, an6—quartz). In that case reliable method of identifying the given grains in SEM is EDS analysis. However, some grains may have such a characteristic shade of gray that they can be easily distinguished from other. Clusters of unreacted cement (Figure A2, an1) stand out as light “grains” against other mineral grains (Figure A2, an3, an4, an6) or mixture of clay minerals (Figure A2, an5) constituting the dark grey background of the sample. The most vivid white grains present in the sample belong to naturally occurring grains of iron oxides and/or barite (Figure A2, an2). Unreacted cement, in addition to the characteristic brightness threshold, also has a relatively characteristic texture, it occurs as a filling the background of the sample, bonding clusters of clay minerals. In contrast, iron oxides, barite or other rock-forming minerals occur as single grains embedded in clay.

## References

[1] Hall M.R., Swaney W. (2012). European modern earth construction. Modern Earth Buildings. Materials, Engineering, Construction and Applications.

[2] Piasecki M., Kozicki M., Firląg S., Goljan A., Kostyrko K. (2018). The Approach of Including TVOCs Concentration in the Indoor Environmental Quality Model (IEQ)-Case Studies of BREEAM Certified Office Buildings. Sustainability.

[3] Anysz H., Narloch P. (2019). Designing the composition of cement stabilized rammed earth using artificial neural networks. Materials.

[4] Minke G. (2006). Building with Earth: Design and Technology of a Sustainable Architecture.

[5] Narloch P.L., Woyciechowski P., Jęda P. (2015). The influence of loam type and cement content on the compressive strength of rammed earth. Arch. Civ. Eng..

[6] Ciancio D., Jaquin P., Walker P. (2013). Advances on the assessment of soil suitability for rammed earth. Constr. Build. Mater..

[7] Consoli N.C., Festugato L., Gravina da Rocha C., Caberlon Cruz R. (2013). Key parameters for strength control of rammed sand–cement mixtures: Influence of types of portland cement. Constr. Build. Mater..

[8] Lina H., Zhenga S., Lourençoa S., Jaquin P. (2017). Characterization of coarse soils derived from igneous rocks for rammed earth. Eng. Geol..

[9] Bui Q.-B., Morel J.-C. (2015). First Exploratory Study on the Ageing of Rammed Earth Material. Materials.

[10] Bui Q.B., Morel J.C., Venkatarama Reddy B.V., Ghayad W. (2009). Durability of rammed earth walls exposed for 20 years to natural weathering. Build. Environ..

[11] Venkatarama Reddy B.V., PKumar P. (2011). Cement stabilised rammed earth. Part A: Compaction characteristics and physical properties of compacted cement stabilised soils. Mater. Struct..

[12] Bui Q.-B. (2017). Assessing the Rebound Hammer Test for Rammed Earth Material. Sustainability.

[13] Bui Q.-B., Morel J.-C., Hans S., Walker P. (2014). Effect of moisture content on the mechanical characteristics of rammed earth. Constr. Build. Mater..

[14] Christopher B., Ciancio D. (2014). Effect of compaction water content on the strength of cement-stabilized rammed earth materials. Can. Geotech. J..

[15] Hall M., Allinson D. (2009). Influence of cementitious binder content on moisture transport in stabilised earth materials analysed using 1-dimensional sharp wet front theory. Build. Environ..

[16] Hall M., Allinson D. (2008). Assessing the moisture-content-dependent parameters of stabilised earth materials using the cyclic-response admittance method. Energy Build..

[17] Pagacz J., Pielichowski K. (2009). Preparation and characterization of PVC/montmorillonite nanocomposites–A review. J. Vinyl Addit. Technol..

[18] Arrigoni A., Grillet A.-C., Pelosato R., Dotelli G., Beckett C., Woloszyn M., Ciancio D. (2017). Reduction of rammed earth’s hygroscopic performance under stabilisation: An experimental investigation. Build. Environ..

[19] Grabowska-Olszewska B. (1998). Geologia Stosowana.

[20] Punmia B.C., Jain A.K., Jain A.K. (2005). Soil Mechanics and Foundations.

[21] Reddy B.V. (2012). Stabilised soil blocks for structural masonry in earth construction. Modern Earth Buildings; Materials, Engineering, Construction and Applications.

[22] Reddi L.N., Jain A.K., Yun H.-B. (2012). Soil materials for earth construction properties, classification and suitability testing. Modern Earth Buildings; Materials, Engineering, Construction and Applications.

[23] Houben H., Guillaud H. (1994). Earth Construction: A Comprehensive Guide.

[24] Yanguatin H., JTobón J., JRamírez J. (2017). Pozzolanic reactivity of kaolin clays, a review. Revista Ingenieria de Construccion.

[25] Grim R.E., Press F. (1968). Clay Mineralogy, International Series in the Earth and Planetary Sciences.

[26] Karpinski B., Szkodo M. (2015). Clay minerals–mineralogy and phenomenon of clay swelling in oil & gas industry. Adv. Mater. Sci. Eng..

[27] Kaczyński R., Grabowska-Olszewska B. (1997). Soil mechanics of the potentially expansive clays in Poland. Appl. Clay Sci..

[28] Seed H.B., Woodward R.J., Lundgren R. (1962). Prediction of Swelling Potential for Compacted Clays. Trans. Am. Soc. Civ. Eng..

[29] Oliver M., Mesbah A. The earth as a material. Proceedings of the International Symposium on Modern Earth Construction.

[30] Borkowska M., Smulikowski K. (1973). Mineraly Skałotworcze.

[31] Narloch P., Hassanat A., Tarawneh A.S., Anysz H., Kotowski J., Almohammadi K. (2019). Predicting Compressive Strength of Cement-Stabilized Rammed Earth Based on SEM Images Using Computer Vision and Deep Learning. Appl. Sci..

[32] Narloch P.L. (2017). Ziemia Ubijana Stabilizowana Cementem Jako Konstrukcyjny Materiał Budowlany w Klimacie Umiarkowanym. Ph.D. Thesis.

[33] Narloch P.L., Lidner M., Kunicka M., Bielecki M. (2015). Flexural tensile strength of construction elements made out of cement stabilized rammed earth. Procedia Eng..

[34] Narloch P.L., Woyciechowski P.P., Dmowska E., Halemba K. (2015). Durability assessment of monolithic rammed earth walls. Arch. Civ. Eng..

[35] Kariyawasam K.K.G.K.D., Jayasinghe C. (2016). Cement stabilized rammed earth as a sustainable construction material. Constr. Build. Mater..

[36] Rempel A.W., Rempel A.R. (2019). Frost Resilience of Stabilized Earth Building Materials. Geosciences.

[37] Firląg S., Piasecki M. (2018). NZEB Renovation Definition in a Heating Dominated Climate: Case Study of Poland. Appl. Sci..

[38] Rahmat M.R., Ismail N. (2018). Effect of optimum compaction moisture content formulations on the strength and durability of sustainable stabilised materials. Appl. Clay Sci..

[39] Hall M., Djerbib Y. (2004). Rammed earth sample production: Context, recommendations and consistency. Constr. Build. Mater..

[40] NZS 4298 (1998). Materials and Workmanship for Earth Buildings.

[41] PN-B-04481:1988 (1988). Grunty Budowlane—Badania Próbek Gruntu.

[42] BS EN 12390-3 (2019). Testing Hardened Concrete; Compressive Strength of Test Specimens.

